# RCA-LF: Dense Light Field Reconstruction Using Residual Channel Attention Networks

**DOI:** 10.3390/s22145254

**Published:** 2022-07-14

**Authors:** Ahmed Salem, Hatem Ibrahem, Hyun-Soo Kang

**Affiliations:** 1 School of Information and Communication Engineering, College of Electrical and Computer Engineering, Chungbuk National University, Cheongju 28644, Korea; ahmeddiefy@chungbuk.ac.kr (A.S.); hatem@chungbuk.ac.kr (H.I.); 2 Electrical Engineering Department, Faculty of Engineering, Assiut University, Assiut 71515, Egypt

**Keywords:** light field reconstruction, based view synthesis, angular super-resolution, channel attention network

## Abstract

Dense multi-view image reconstruction has played an active role in research for a long time and interest has recently increased. Multi-view images can solve many problems and enhance the efficiency of many applications. This paper presents a more specific solution for reconstructing high-density light field (LF) images. We present this solution for images captured by Lytro Illum cameras to solve the implicit problem related to the discrepancy between angular and spatial resolution resulting from poor sensor resolution. We introduce the residual channel attention light field (RCA-LF) structure to solve different LF reconstruction tasks. In our approach, view images are grouped in one stack where epipolar information is available. We use 2D convolution layers to process and extract features from the stacked view images. Our method adopts the channel attention mechanism to learn the relation between different views and give higher weight to the most important features, restoring more texture details. Finally, experimental results indicate that the proposed model outperforms earlier state-of-the-art methods for visual and numerical evaluation.

## 1. Introduction

Light fields (LF) record 3D scenes into uniform and dense image samples. These images contain spatial and angular information about the 3D scenes. As a result, many applications have developed and benefited greatly from this huge amount of information, such as de-occlusion [1,2], depth-sensing [3,4,5], saliency detection [6], and salient object detection [7]. In addition, LF could be promising to ease other applications such as the fruit-picking robot, where a robot traverses a whole field and harvests on its own [8,9]. LF images are caught using portable cameras or camera arrays in most situations. In order to use array cameras, several cameras are required, which is an expensive and laborious process [10]. A practical solution for capturing LF images with portable cameras can be provided by inserting a microlens array in front of the image sensor [11,12]. Despite the advantages of this solution, it comes with a major drawback: poor sensor resolution. Therefore, obtaining LF images with high spatial and angular resolution is difficult.

Recently, several learning-based approaches that considerably enhance the performance of LF reconstruction have been presented. The LF reconstruction challenge reconstructs dense LF images from sparse input views. Previous approaches using the convolutional neural network (CNN) without depth estimation [13,14] can only handle LFs with a small baseline. They explore the connection between the angular and spatial domains but fail to use the epipolar information fully.

Some approaches [15,16] estimate depth maps and warp views to investigate relationships between views. However, the wrongness of the calculated depth map greatly affects how the LF reconstruction turns out. There is another approach to mitigate the effect of limited sensor resolution through LF super-resolution [17,18,19], but this is outside this research’s interest.

This article presents a unique learning-based methodology for rapidly reconstructing a densely sampled LF from a very sparsely sampled LF. Computationally efficient convolutions realize our end-to-end CNN model to understand spatial-angular relationships deeply. We up-sample the sparsely input LF to the required angular size using the bicubic interpolation in the preprocessing stage. The RCA-LF is then deployed to leverage the inherent LF structure in the up-sampled LF images. Notably, our method does not need disparity warping or intensive computations. In addition, it reconstructs a whole LF in a single forward pass. Specifically, we introduce the residual channel attention light field (RCA-LF) structure to solve different LF reconstruction tasks. In our approach, view images are grouped in one stack where epipolar information is available. We use 2D convolution layers to process and extract features from the stacked view images. Our method adopts the channel attention mechanism to learn the relation between different views and give higher weights to the most important features, restoring more texture details.

We propose a new way to process the multi-channel input, which comes from 2D convolution instead of 3D convolution. Two-dimensional convolution takes a single slice as an input and fails to leverage context from adjacent slices. Conversely, 3D convolution overcomes this issue by leveraging the slice context with 3D convolutional kernels, resulting in enhanced performance. However, 3D convolutions have a limited range depending on the kernel size (3 × 3 × 3 kernels can leverage depth information using only three consecutive slices).

In our proposed method, the input has a size of (B, H, W, 49) for the 3 × 3 to 7 × 7 reconstruction task where the 49 represents the number of input channels. For 2D convolution, the number of filters equals (filter_height × filter_width × in_channels × out_channels). Consequently, every output channel is a function of all input channels at each convolution. Adopting this method can fix the limited range issue of the 3D convolution and provide better quality.

The number of input channels is 49 for the 3 × 3 to 7 × 7 reconstruction task. Still, we extract more features on the subsequent convolution layers, meaning more interactions can be identified between the extracted features of input images, restoring more information and details. Because some of the extracted features might contain useless or redundant information, the channel attention mechanism rescales (gives different weights) for these extracted features depending on the information content.

We can summarize the contributions of this article as follows: (1) We adopt a channel attention mechanism to reconstruct LF images. (2) Our method increases the interaction between different LF images by processing LF images as input–output channels of 2D convolutions. (3) We design the RCA-LF to increase the interactions between input–output channels (parallel processing) and decrease the number of blocks (serial processing); hence, it can reconstruct LF images accurately and fast.

## 2. Related Work

### 2.1. LF Representation

**Figure 1:**
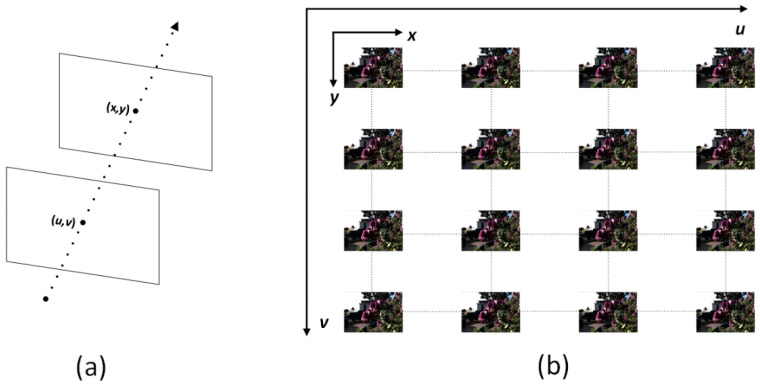
(**a**) The 4D LF representation with two planes’ intersections of light rays. (**b**) LF images.

A wealth of information about the surrounding 3D space is revealed by LF imaging, contrary to traditional imaging methods. The Plenoptic function was initially described using seven variables that determine the view from any possible angle, for all wavelengths of light and at any time, as $P=P\left(\theta ,\varphi ,\lambda ,t,V_{x},V_{y},V_{z}\right)$ [20]. It was then simplified to a 4D description with the intersections of light rays with two planes L=L(u,v,x,y), where (*u*, *v*) and (*x*, *y*) denote the points of intersection with the first and second planes, respectively, as shown in Figure 1 [21].

### 2.2. LF Reconstruction

Many LF reconstruction approaches have been presented. These approaches are classified into three types: traditional, deep learning depth-based, and deep learning non-depth-based approaches.

#### 2.2.1. Traditional Approaches

Wanner and Goldluecke improved the spatial and angular resolutions using the Epipolar Plane Image (EPI) for depth map estimation [22]. However, this variational framework has flaws since the input views only assess the disparity. Another approach was proposed to utilize the Gaussian mixture model for LF denoising, super-resolution, and refocusing [23]. In this approach, the patch prior was designed using the disparity pattern. However, their approach is vulnerable to low-quality LF images. Pujades et al. [24] proposed a novel cost function optimized by a Bayesian formulation to estimate the depth and reconstruct novel views. Chaurasia et al. [25] proposed a novel image-based rendering using superpixels to preserve depth discontinuities. The warped views are blended using a camera and depth information. Zhang et al. [26] proposed an interactive system adopting patch-based methods for LF editing. This technique models the collected images as overlapping layers with varying depths and uses back-to-front layered synthesis. Vagharshakyan et al. [27] utilized the EPIs in the shearlet domain to reconstruct dense images using large baseline-rectified images. Their method provided good results for non-Lambertian scenes of semi-transparent objects.

#### 2.2.2. Deep Learning Depth-Based Approaches

Kalantari et al. [15] suggested decomposing the reconstruction process into disparity and color estimates independently evaluated by the relevant CNN network. Due to their separate reconstruction, connections between novel LF images were overlooked. Another approach was proposed to speed up Kalantari’s method using a predefined CNN [28]. In addition, they proposed the estimation of two disparity maps to provide more accurate results. Shi et al. [16] used two reconstruction modules: pixel reconstruction to handle the occlusions explicitly, and feature reconstruction for high frequencies. However, this method was limited by the need for depth maps. In contrast to the previous methods designed for images with a small baseline, Jin et al. [29] designed a model for images with a large baseline. A CNN was employed to estimate depth maps to wrap input views, and these views were then blended using a SAS CNN [30]. Because the quality of synthesized views is dependent on the accuracy of estimated depth maps, unwanted artifacts often emerge in synthesized views.

#### 2.2.3. Deep Learning Non-Depth-Based Approaches

Most of these methods extract information from EPIs for the reconstruction process. Wu et al. [31] divided the process into low-frequency restoration after a blur operation and high-frequency restoration by inverting the blur operation. However, they did not use the epipolar information efficiently, as they only extracted the EPIs in one direction. Using a CNN, Wu et al. [32] applied a shearing operation to input EPIs to eliminate the effect of significant disparities. Then, they employed a CNN to learn a fusion score. In this method, the authors misused the angular information by using EPIs horizontally or vertically for the reconstruction. In addition, they reconstructed rows and then columns hierarchically, leading to reconstruction error accumulation. Meng et al. [33] proposed an HDDRNet for LF spatial and angular super-resolution employing a high dimensional CNN. Although they used the provided angular information efficiently, employing the 4D convolutions, this was at the expense of model complexity. Mildenhall et al. [34] proposed reconstructing multi-plane images from input views and then blending them to reconstruct novel views. Wang et al. [35] used EPI and EPI stacking to create a pseudo-4D CNN. They used EPI structure-preserving loss to increase reconstruction quality. They wasted angular data by only using horizontal or vertical EPI stacks. Hu et al. [14] proposed LF reconstruction with hierarchical feature fusion. SAS layers were employed to extract features from 4D LF images, while the U-Net structure was adopted to generate both semantic and local feature representation. They integrated these two structures and proposed a U-SAS module to enable the extraction of spatial features and the correlation of SAIs. In addition, they adopted an enlarged patch size when training for the integrated information of objects. Liu et al. [36] proposed to extract EPI information in a horizontal, vertical, and angular manner to reconstruct LF images. However, each branch was processed alone, which affected the final quality. Zhang et al. [37] reconstructed LF images employing 2D and 3D CNNs on horizontal and vertical EPIs. However, they neglected the angular LF information, slightly affecting the final reconstruction quality. Salem et al. [38] mapped the LF reconstruction problem from the 4D into the 2D domain by transforming the 4D LF into a 2D raw LF image to ease the reconstruction. They provided satisfactory reconstruction quality using a model inspired by the RCAN [39,40]. Still, they used a heavy model, which affected the reconstruction time.

## 3. Methodology

### 3.1. Problem Formulation

**Figure 2:**
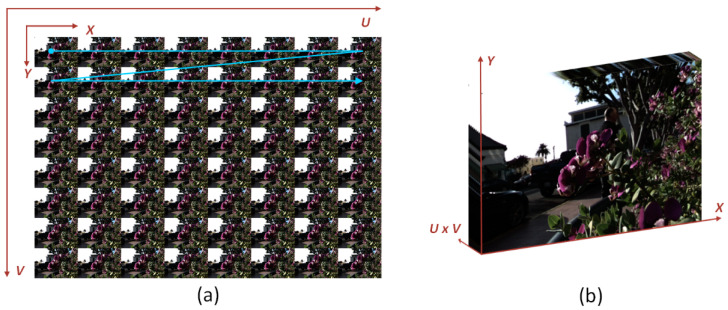
(**a**) The 4D LF representation as a 2D array of view images. (**b**) The 3D LF view image stack.

We can consider the LF images as a 2D array of view images, as shown in Figure 2a. These images have (*H*, *W*) and (*U*, *V*) spatial and angular resolutions. Our goal is to reconstruct dense LF images from their sparse input counterparts. Assume $L^{LR}\in R^{H\times W\times u\times v}$ represents the sparse input views with angular resolution (*u*, *v*). Using the *LR* input, our RCA-LF network can reconstruct a dense output $L^{HR}\in R^{H\times W\times U\times V}$ with (*U*, *V*) angular resolution. Before applying the *LR* input images, we up-sample the sparse input EPIs to the required output size utilizing the Bicubic interpolation to generate ${\tilde{L}}^{LR}\in R^{H\times W\times U\times V}$. The last step before applying the *LR* input to the network is to rearrange it from the 4D representation ${\tilde{L}}^{LR}\in R^{H\times W\times U\times V}$ into the 3D representation ${\tilde{L}}^{LR}\in R^{H\times W\times UV}$, as shown in Figure 2b. We reconstruct the 3D ${\tilde{L}}^{LR}$ by stacking the view images in row-major order as indicated by the blue line in Figure 2a.

### 3.2. Network Architecture

**Figure 3:**
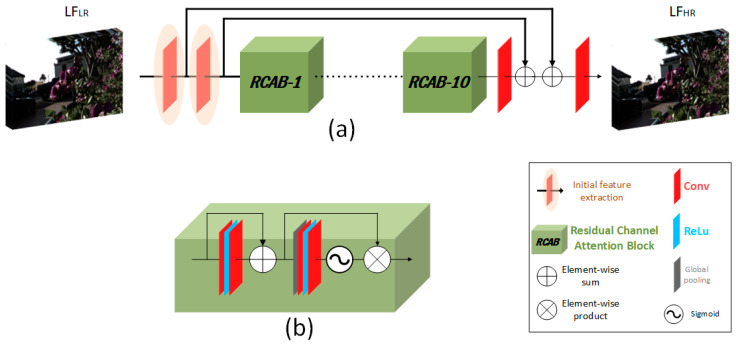
(**a**) Overview of the proposed RCA-LF network structure. (**b**) Implementation details of the residual channel attention block (RCAB).

We designed our network similarly to the RCAN network [39]. In terms of functionality, our model can be divided into primary feature extraction, deep feature extraction, and final output restoration, as shown in Figure 3a. The primary feature extraction is implemented using two convolutional layers (Conv). Each Conv is followed by a long skip connection to bypass the low-frequency components to the output part, allowing the network to concentrate on high-frequency component extraction. The deep feature extraction is implemented using ten residual channel attention blocks (RCAB), as shown in Figure 3b. The final part is implemented by summing the primary extracted features with the deep extracted features to reconstruct the final output.

This is unlike the RCAN method, in which the input is a single-channel input. Then, channel-wise features are extracted from the input to be processed through the network. The input in our method is a stack of U×V images (multi-channel input), where (*U*, *V*) is the angular resolution. Then, more channel-wise features are extracted with the extraction ratio e to be e×U×V. The RCAB is the main component of our network, as the RCA-LF consists of ten RCABs. The RCAB is a residual block (RB) with an integrated channel attention mechanism (CA). The first part of the RCAB, RB, is built by cascading two Conv layers with an activation function (ReLU) with a skip connection.

The CA is adopted to allow the network to treat the extracted channel-wise features unequally and concentrate on the crucial features. A global average pooling is used to shrink the intermediate C feature map of size H×W into 1×1 to obtain the initial channel-wise statistics to determine which channels are more important. These channel statistics may be considered a collection of local descriptors to express the full-view stack [41]. A Conv then down-samples these initial statistics with a reduction ratio of r. A Conv up-samples these statistics with the same reduction ratio after being activated by ReLU, as shown in Figure 3 in [39]. Finally, a gate mechanism is applied to learn the nonlinear interactions between channels and the non-exclusive mutual relationship. The gate mechanism is applied with a sigmoid function to obtain the final channel-wise statistics.

### 3.3. Implementation Details

The luminance component is only used to train the RCA-LF network, while the EPIs of the chrominance components are up-sampled with the Bicubic interpolation. We trained our network to map the *LR* input images to the HR LF output images by minimizing the *L*_1_ loss and optimizing the Adam optimizer with its default parameters [42]. The *L*_1_ loss is defined as follows when a training set has *N* combinations of input and counterpart ground-truth pictures:

$$L_{1}=\frac{1}{N}\sum_{i=1}^{N}\left|L_{HR}^{i}-f\left(L_{LR}^{i}\right)\right|$$

f() represents the function responsible for mapping the *LR* input into the HR output and is implemented by the RCA-LF network. All the Conv layers used were of size 3×3 with zero padding, except for the Conv layers used for the CA, which were of size 1×1. Both the extraction ratio e and the reduction ratio were set to 8. We trained the network with patches of size 32×32 and a batch size of 128. We started the training with an initial learning rate of $10^{-4}$ and decreased it exponentially by 0.1 every 100 epochs while we trained the network for 150 epochs. We used 100 full LF images to train our network [15,43], using TensorFlow [44] on an NVIDIA GeForce RTX 3090 GPU. PSNR and SSIM were used as reconstruction quality assessment indicators.

## 4. Experiments and Discussion

**Figure 4:**
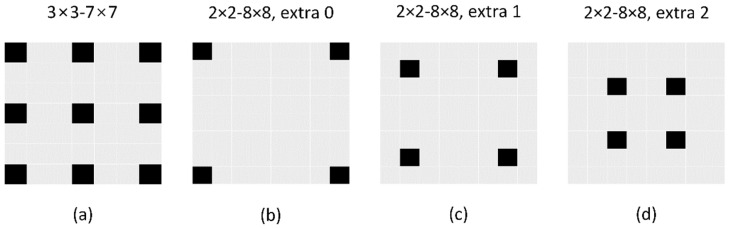
The input–output relationship for different LF reconstruction tasks. (**a**) 3 × 3–7 × 7. (**b**) 2 × 2–8 × 8, extra 0. (**c**) 2 × 2–8 × 8, extra 1. (**d**) 2 × 2–8 × 8, extra 2.

We conducted comprehensive experiments to validate the effect of the proposed RAC_LF network. We compared the RCA_LF numerically and visually with state-of-the-art methods using real-world LF images. We used 30 LFs from the 30 scenes dataset [15], 31 LFs from the refractive and reflective surfaces dataset [43], and 43 LFs from the occlusions dataset [43]. The average PSNR and SSIM [45] over the reconstructed LF luminance were used for the numerical comparison. We compared the RCA_LF over two interpolation tasks (2 × 2–8 × 8 and 3 × 3–7 × 7) and two extrapolation tasks (2 × 2–8 × 8 extrapolations 1 and 2), as shown in Figure 4.

**Figure 5:**
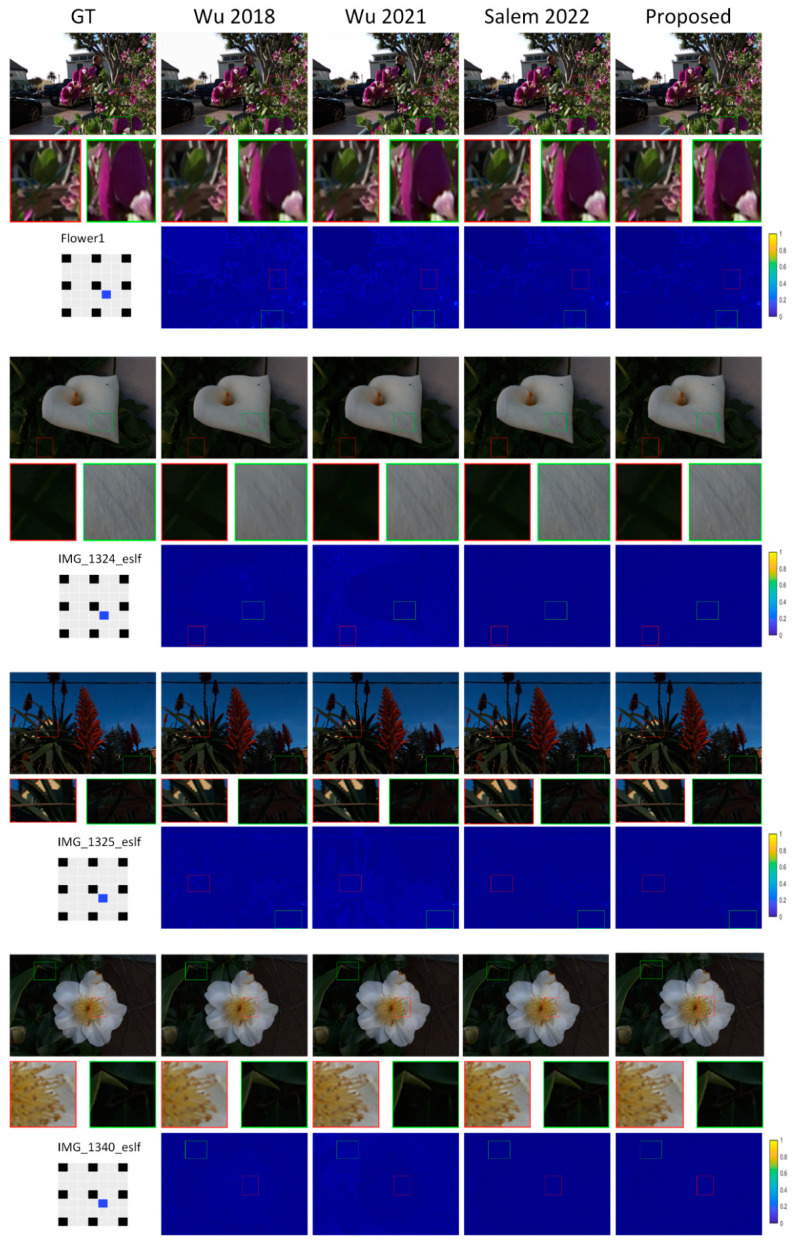
Visual comparison of the proposed model to reconstruct 7 × 7 views out of 3 × 3.

**Figure 6:**
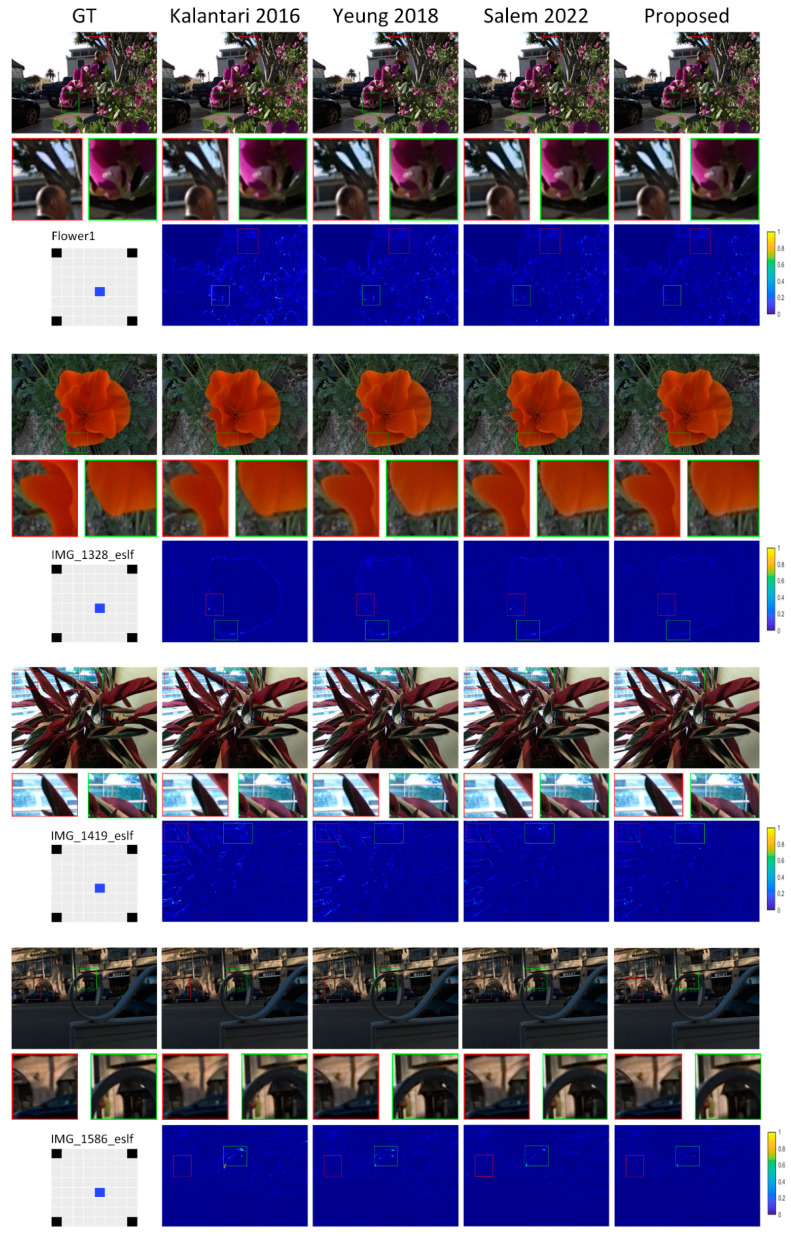
Visual comparison of the proposed model to reconstruct 8 × 8 views out of 2 × 2: extrapolation 0.

**Figure 7:**
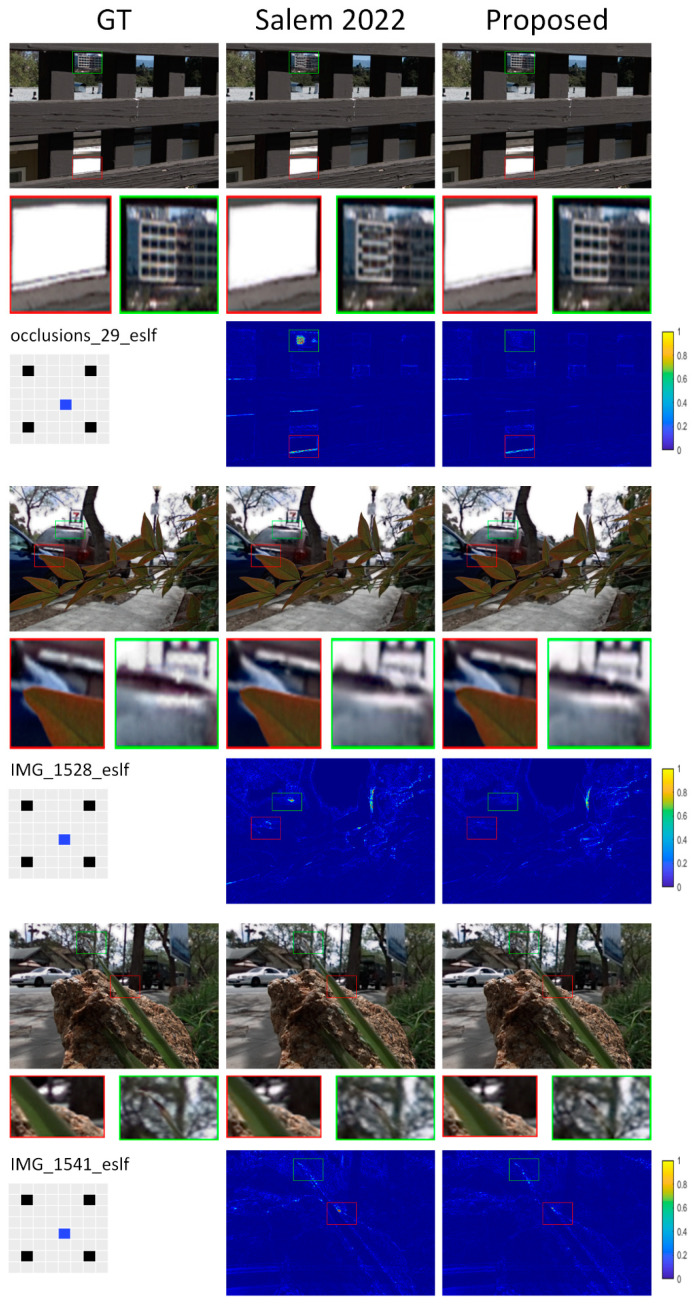
Visual comparison of the proposed model to reconstruct 8 × 8 views out of 2 × 2: extrapolation 1 at an interpolated view.

**Figure 8:**
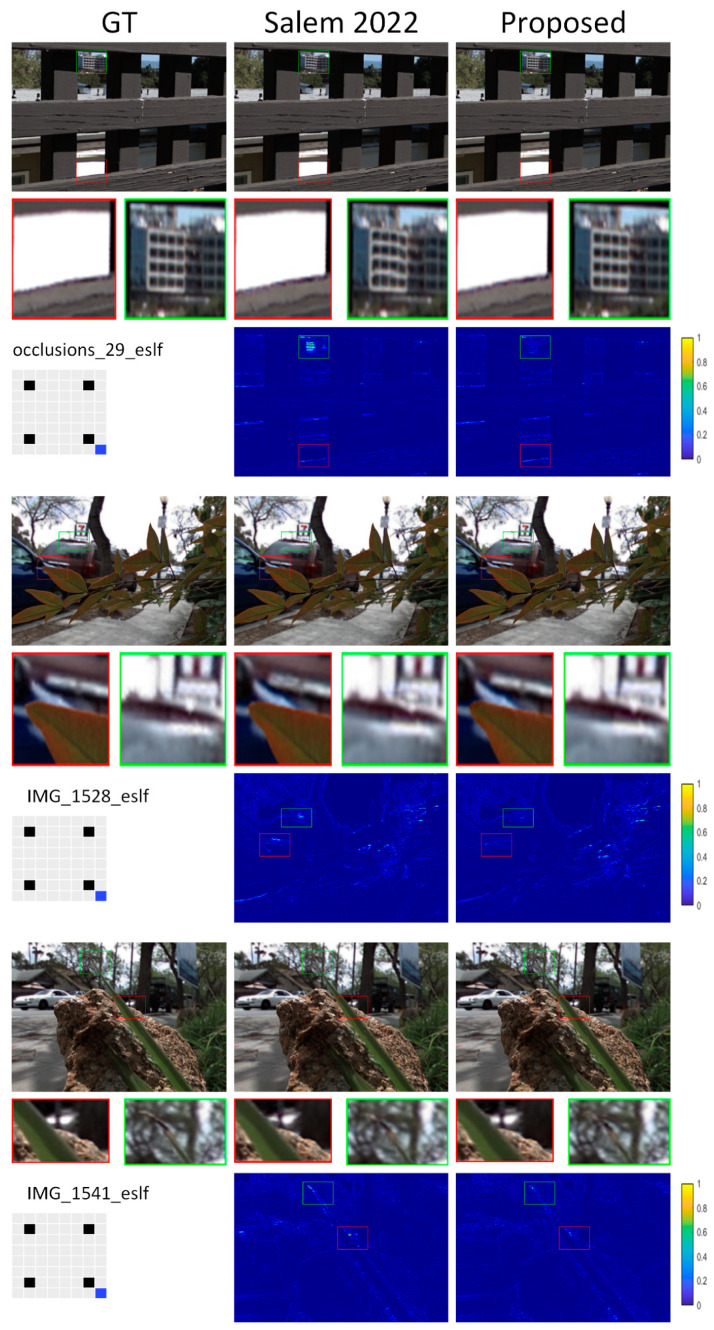
Visual comparison of the proposed model to reconstruct 8 × 8 views out of 2 × 2: extrapolation 1 at an extrapolated view.

Table 1, Table 2, Table 3 and Table 4 present numerical data indicating the proposed approach’s effectiveness. Numerical comparisons are provided regarding peak-signal-to-noise ratio (PSNR) and the structural similarity index (SSIM) [45]. Figure 5, Figure 6, Figure 7 and Figure 8 show a visual contrast highlighting our model’s ability to recreate high-quality images with sharper edges around object boundaries, even in obscured areas and against complex backgrounds. However, we attribute the significant improvement in our model results to: (1) 3D representation (LF view stack), allowing the network to model and understand relations between different LFs; (2) the channel attention mechanism, which played an important role by allowing the network to concentrate on the crucial features.

### 4.1. Different Reconstruction Tasks

#### 4.1.1. Task 3 × 3–7 × 7

Wu et al. [31] underutilized angular data, using EPIs in just one direction. Utilizing EPIs in horizontal and vertical dimensions subsequently yielded superior outcomes. Nonetheless, they hierarchically up-sampled LF, increasing error accumulation on the most recently reconstructed views. In addition, they proposed a second paradigm based on sheared EPIs [32]. In particular, low-angular-resolution EPIs were sheared before being up-sampled to the necessary angular resolution. The up-sampled EPIs with various shearing methods were fused by learning fusion scores using a CNN. Liu et al. [36] used angular information more effectively than earlier techniques, yet this was insufficient since they only employed one EPI stack in each direction. Zhang et al. [37] used micro-lens pictures and view image stacks to investigate further LF data. Salem et al. [38] used the raw LF representation to ease the reconstruction process. In addition, they initialized the input image using the nearest view initialization method. However, this method had a limitation for some reconstruction tasks. Additionally, it affected the quality of the final image.

**Table 1:** The proposed model’s numerical comparison (PSNR/SSIM) model to reconstruct 7 × 7 out of 3 × 3 views.

Dataset	Wu [31]	Wu [32]	Liu [36]	Zhang [37]	Salem [38]	Proposed
30 Scenes	41.40/0.980	43.592/0.986	44.86/0.991	45.68/0.992	45.96/0.991	46.41/0.992
Reflective	42.19/0.974	43.092/0.977	44.31/0.980	44.92/0.982	45.45/0.983	45.73/0.984
Occlusions	37.25/0.925	39.748/0.948	40.16/0.957	40.80/0.955	41.21/0.957	41.41/0.951
Average	40.28/0.959	42.14/0.971	43.11/0.976	43.80/0.976	44.21/0.977	44.51/0.976

#### 4.1.2. Task 2 × 2–8 × 8, Extrapolation 0

Both Kalantari et al. [15] and Shi et al. [16] generate new views by distorting the input views by their assessed disparity/depth. On the other hand, depth estimation and warping are challenging, particularly for LF pictures with a tiny depth difference, making it possible for images to be flawed and seem out of place. Due to Yeung et al.’s disregard for the connections between distinct views, their approach generates false shadows and ghosting artifacts at the borders of reconstructed views [46].

#### 4.1.3. Task 2 × 2–8 × 8, Extrapolation 1, 2

Reconstructing 8 × 8 out of 2 × 2 views is a challenging task due to the sparseness of the input views. Yeung et al. [46] observed that the reconstruction quality of the center views is much worse than that of the views located near the input views. Because the center view is the farthest distance from any input views, inferring the details with greater accuracy presents the biggest problem. Therefore, they proposed different combinations of interpolation and extrapolation to reconstruct LF images. As a result, the average distance from all the novel views is shorter than before, increasing the reconstruction quality of the center views. Most available algorithms are optimized for interpolation tasks and cannot predict extrapolated views. That is why ghosting and artifacts often appear around thin structures and occluded regions. Extrapolation is more challenging than interpolation because certain portions of the reconstructed views are not present in the input. In addition, it cannot keep the slopes of the lines in the reconstructed EPIs the same. It is challenging to devise a method for dealing with different relationships between input and output views. However, the task becomes more feasible and efficient with our proposed approach.

**Table 2:** The proposed model’s numerical comparison (PSNR/SSIM) to reconstruct 8 × 8 out of 2 × 2 views: extrapolation 0.

Dataset	Wu [31]	Kalantari [15]	Shi [16]	Yeung [46]	Zhang [37]	Salem [40]	Proposed
30 Scenes	35.25/0.928	40.11/0.979	41.12/0.985	41.21/0.982	41.98/0.986	42.33/0.985	42.69/0.986
Reflective	35.15/0.940	37.35/0.954	38.10/0.958	38.09/0.959	38.71/0.962	38.86/0.962	39.45/0.967
Occlusions	31.77/0.881	33.21/0.911	34.41/0.929	34.50/0.921	34.76/0.918	34.69/0.922	35.41/0.928
Average	34.06/0.916	36.89/0.948	37.88/0.957	37.93/0.954	38.48/0.955	38.62/0.956	39.18/0.960

**Table 3:** The proposed model’s numerical comparison (PSNR/SSIM) to reconstruct 8 × 8 out of 2 × 2 views: extrapolation 1.

Dataset	Yeung [46]	Zhang [37]	Salem [40]	Proposed
30 Scenes	42.47/0.985	43.57/0.989	43.76/0.988	44.26/0.989
Reflective	41.61/0.973	42.33/0.975	42.44/0.974	43.16/0.979
Occlusions	37.28/0.934	37.61/0.937	37.93/0.948	38.47/0.943
Average	40.45/0.964	41.17/0.967	41.38/0.970	41.96/0.970

**Table 4:** The proposed model’s numerical comparison (PSNR/SSIM) to reconstruct 8 × 8 out of 2 × 2 views: extrapolation 2.

Dataset	Yeung [46]	Zhang [37]	Salem [40]	Proposed
30 Scenes	42.74/0.986	43.41/0.989	43.43/0.987	43.92/0.989
Reflective	41.52/0.972	42.09/0.975	42.26/0.975	42.81/0.978
Occlusions	36.96/0.937	37.60/0.944	37.91/0.945	38.25/0.935
Average	40.41/0.965	41.03/0.969	41.20/0.969	41.66/0.967

### 4.2. Reconstruction Time

Table 5 presents the average run-time to reconstruct a full LF image for the first task: 7 × 7 out of 3 × 3 views. We tested our model on an NVIDIA Geforce RTX 3090. The proposed model can reconstruct LF images faster due to its highly parallel design.

Wang et al. [47] consume a lot of time as they do not reconstruct the entire scene in one feedforward pass. Instead, they reconstruct rows and then columns hierarchically. Yeung et al. [46] and Liu et al. [36] used MATLAB to build their code, which contains many time-consuming reshaping operations. Compared to Salem et al. [38], they used 15 residual blocks (RBs) compared to the 10 RBs in our proposed work. In addition, they process LFs in the raw representation of size 7*H* × 7*W* compared to *H* × *W* in our implementation.

**Table 5:** Average run-time to reconstruct 7 × 7 out of 3 × 3 views.

	Wang [47]	Yeung [46]	Liu [36]	Salem [38]	Proposed
Run-Time	5.74 s	4.58 s	2.45 s	1.911 s	0.686 s

### 4.3. Ablation Study

**Figure 9:**
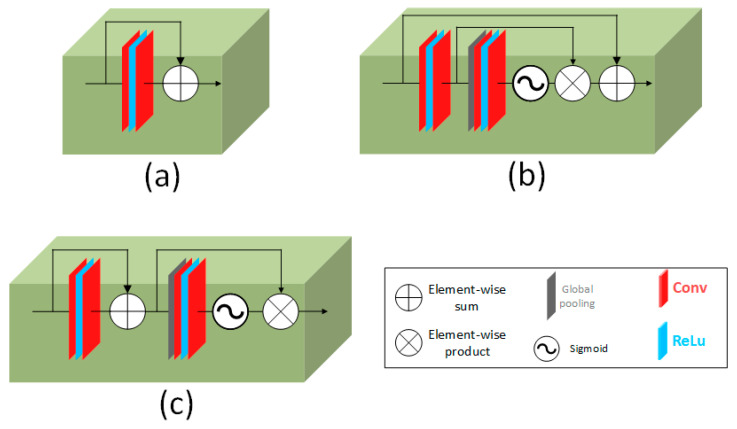
Different designs of the residual channel attention block (RCAB); (**a**) simple residual block (RB) without the channel attention mechanism; (**b**) the proposed RCAB with the CA integrated inside [39]; (**c**) the proposed RCAB with a CA separated from the RB.

We compared three different architectures to validate the effect of the channel attention (CA) mechanism on the reconstruction process. Numerical comparison is presented in Table 6, where the first row indicates the simplest case without applying the CA mechanism. The second row gives the results for the block that is the same as the one proposed in [39] with the CA integrated inside the RCAB. The final row gives the results for the proposed block with the CA separated from the RB, as shown in Figure 9.

**Table 6:** Investigating the channel attention mechanism (CA) effect on the proposed architecture.

Model 3 × 3–7 × 7	30 Scenes	Reflective	Occlusions	Average
No CA	44.86/0.990	44.74/0.981	40.06/0.951	43.22/0.974
CA inside RB	46.20/0.992	45.71/0.984	41.35/0.954	44.42/0.976
CA separated from RB	46.41/0.992	45.73/0.984	41.41/0.951	44.51/0.976

## 5. Future Work

In this paper, we present a method for reconstructing light field images. The proposed method is characterized by its applicability to all reconstruction tasks for LF images with a small baseline. Although this model is efficient, it fails to reconstruct LF images with a broad baseline. In addition, it sometimes fails to reconstruct parts of the scenes with complex backgrounds or contains severe reflections. Therefore, we are trying to develop a method capable of reconstructing complex scenes and scenes with a broad baseline.

## 6. Conclusions

This research proposes an effective learning-based paradigm for increasing the angular resolution of LF images. We up-sampled input EPIs to the required angular size, which allows our network to be used for any reconstruction task. In addition, this allowed the network to comprehend and accurately represent the connection since the input and output were of the same size. Finally, we adopted the channel attention mechanism to help the network to concentrate on the important features by assigning higher weights. The proposed RCA_LF network reconstructs LF images by mapping the up-sampled low-resolution images into high-resolution 3D LF volumes. The RCA_LF outperforms other state-of-the-art methods in reconstructing LF images with a small baseline.

## Data Availability

The datasets used in this paper are public datasets. We also provide the proposed method’s training and evaluation codes at: https://github.com/ahmeddiefy/RCA-LF, which was created (accessed on 24 May 2022).
